# 3D Indoor Scene Reconstruction and Layout Based on Virtual Reality Technology and Few-Shot Learning

**DOI:** 10.1155/2022/4134086

**Published:** 2022-03-24

**Authors:** Huanmei Li

**Affiliations:** School of Art, Taiyuan University of Science and Technology, Taiyuan, Shanxi 030021, China

## Abstract

Indoor three-dimensional layout has a strong application background, such as virtual office three-dimensional layout planning, museum three-dimensional layout planning, and cave scene three-dimensional layout planning, which have been widely used in telecommuting, education, tourism, and other industries. In view of this, this paper proposes an indoor landscape reconstruction method based on VR (virtual reality) and draws indoor landscape information and images by using VR technology to generate an indoor landscape reconstruction panorama. A model is established to correct the distance error and reflectivity error of depth image, improve the accuracy of the depth image, and finally improve the accuracy of three-dimensional indoor scene TDR (three-dimensional reconstruction). In the process of optimizing layout, the Monte Carlo sampling method is used based on the Markov chain, and constraints are used as density functions to guide layout sampling and generate a number of reasonable scene layout suggestions in the iterative process of the sampler. Experiments show that this method can provide scientific and reasonable guidance to users' scene layout and help them complete the furniture layout quickly.

## 1. Introduction

With the vigorous development of VR (virtual reality) technology and the continuous emergence of new devices, TDR (three-dimensional reconstruction) has become a hot research topic in the field of computer vision [1]. The main task is to build a three-dimensional model of the real physical world based on the data collected by various sensors, using mathematical tools such as multiview geometry, probability statistics, and optimization theory, and building a bridge between the real world and the virtual world [2, 3]. The design inspiration of three-dimensional indoor scenes often comes not from simple imagination but from the backwardness of existing scene designs, and there happen to be a large number of realistic scene images on the Internet. In practice, when collecting large amounts of scene image data, such as crowded shopping malls, it is inevitable that dynamic objects will appear, which greatly limits the application of the TDR algorithm in real life [4]. Three-dimensional layout planning of indoor scenes is one of the key research contents in the field of computer vision and an important part of autonomous navigation and unknown environment model reconstruction of mobile robots.

At present, three-dimensional point cloud data acquisition methods are based on a three-dimensional laser scanner and RGB-D camera. The former point cloud based on laser scanning is of high quality, less influenced by the surrounding environment, and is usually used in outdoor large-scale scenes. Because the equipment is complex to operate, inconvenient to carry, and expensive, which usually reaches millions of levels, it is not convenient to collect indoor point clouds [5, 6]. The 3D point cloud reconstruction method based on vision camera mainly uses a multiview stereo geometry algorithm [7]. The motion recovery structure algorithm is an important off-line TDR method that uses several images of a scene to recover the pose of each exposure point and create a three-dimensional point cloud at the same time. It is characterized by time-consuming calculations, but the reconstruction results are extremely accurate [8]. It is necessary to develop an information-based interior color design method based on computer VR technology in order to help users better understand the effect of interior color design and to make it easier for designers to create interior color designs that are more in line with customers' wishes and needs. As a result, the current research hotspot is holographic image based on laser technology, and the research foundation is model building of 3D layout planning of 3D indoor scenes. Data collection, automation degree, model building range, and other issues plague traditional model building of 3D layout planning of indoor scenes [9].

At present, the application in 3D indoor scene design is mainly done by VR technology, which has a good reconstruction effect for noisy animated scene images, and also helps to enhance the realism and immersion of scene design, so as to realize the optimal design of 3D indoor scene model. With the development of deep learning research, some researchers have also proposed an end-to-end method based on deep learning, which is used to directly infer indoor 3D layout from image data. This kind of innovative method is very attractive, but the results often lack accuracy and rigor. Compared with traditional methods, there are still some big defects in practical application. In order to facilitate the reasonable reconstruction of 3D indoor scenes, this paper proposes a brand-new 3D scene modeling method based on VR technology, which mainly solves the two core problems of restoring 3D scenes from a single picture and optimizing the layout of scenes. Based on the solutions of these two problems, the corresponding prototype system of picture scene restoration and layout is designed and implemented. The research in this paper not only makes it convenient for users to get pictures from the Internet and restore picture scenes quickly with little interaction but also puts forward diversified layout suggestions on this basis, which enriches the established 3D scenes.

## 2. Related Work

TDR of the three-dimensional indoor scene based on RGB-d sensor spans many fields such as computer vision and robotics. The author of [10] analyzes the calibration of the Kinect v1 sensor and analyzes its depth image information in terms of accuracy and resolution. This paper studies how to use Kinect v1 to build a dense 3D map of the indoor environment for robot navigation, semantic mapping, and remote presentation. The author of [11] studied the physical measurement principle and capability of TOF technology used in the depth image of Kinect v2 sensor and its error source analysis and put forward a new calibration method of Kinect v2 sensor. The experiment verified that the calibration effect of the two open-source calibration methods was improved, and the reconstruction of a single image was effectively simplified by introducing appropriate interaction. These methods mostly started from the image's own information and realized the geometric reconstruction of a single image by interactively specifying the vanishing point information and any variables, but there were some limitations. The data-driven suggestion method is introduced into 3D modeling in [12], allowing artists to easily use existing 3D models for innovation. To achieve the idea of redesigning a 3D model, their method combines graphics, retrieval, and connection technology among graphics. The improved random sampling consistency algorithm in [13] can effectively eliminate outliers in feature point matching, allowing the SLAM (simultaneous localization and mapping) algorithm to maintain its high robustness in a dynamic environment. The author of [14] proposes Dyna SLAM, which uses a Mask R-CNN network to identify and segment dynamic objects based on prior information, and then repairs the background after removing the dynamic objects. It not only removes dynamic objects but also repairs the background area that has been blocked by dynamic objects and accurately reconstructs the map of pure static scenes. The author of [15] predicted the boundary characteristics of the cavity area using a convolution neural network model combined with a color image and a depth image and realized the filling of the cavity area through function optimization. This method works well and preserves details, but it is heavily influenced by the training data set and takes a long time to learn.

Three-dimensional point cloud is the most basic representation method of three-dimensional scenes, and furthermore, algorithms are needed to mine the scene structure information and semantic information contained in it. The characteristics of buildings in the indoor virtual environment are different from those in the outdoor environment. When building in the indoor environment, the density of the 3D indoor scene needs to be considered. This paper presents a method of constructing solid geometry based on 2D, which can build a more complex indoor layout in layers. The author of [16] focuses on automatic methods to reconstruct the basic semantic information of interior layout, including walls, doors, and windows. The author of [17] proposed a plan reconstruction method based on the Manhattan world hypothesis, so as to reconstruct the visual layout of the room. The author of [18] also proposed an indoor structure reconstruction method based on the Manhattan world hypothesis. The image method is also used in [19] to synthesize the model's facade, and the use of texture allows users to see the real street scene. A data-driven method for generating the room layout of the building plane was proposed in [20]. 120 architectural schemes were used in their study to train a Bayesian network to determine the relationship between rooms. The author of [21] encapsulates architectural design knowledge into an expert system, allowing it to assess the evaluated architectural plane in accordance with government regulations and interior design guidance, and then provides reasonable revision opinions. A similar method for furniture layout is used in [22]. Every furniture object will be connected to its parent, and the constraint relationship between parent and child objects will be specified ahead of time. However, as the number of furniture models in the model library grows, the relationship between parent and child models must be specified on a regular basis, which is a time-consuming task.

## 3. Research Method

### 3.1. 3D Indoor Scene Reconstruction Based on VR Technology

Color camera of RGB-D sensor collects color images, while infrared camera collects depth images. They all calibrate based on the pinhole model and establish the transformation relationship from 3D world coordinates to 2D image coordinates. Before using any camera, it is necessary to calibrate it, and so is the RGB-D sensor. It is necessary to get the internal and external parameters and distortion coefficient of the RGB-D sensor.

RGB-D image hardly changes in scenes such as changing illumination and complex background, but the image is affected by environmental noise and equipment noise when shooting, so it is impossible to encode the depth of every point in space. To improve the rationality of segmentation, the following types of points are removed:

Boundary point: in a neighborhood of image pixels, the local normal consistency is used to judge boundary points, which mainly include object edges, high curvature edges, and occluded scene edges.

Missing points of data [2]: when acquiring scene data, there are some areas that are not perceived, which leads to data missing.

According to the above definition, the boundary area is excluded in a neighborhood of each pixel, and the calculation formula is

(1) $$\begin{array}{c} A\left(c\right)=F\;\sin\left(a,b\right), \end{array}$$

where sin(*a*, *b*) represents a pixel; *F* is the image boundary area.

The input image is over-segmented using an image segmentation algorithm [23, 24]. Although the scene cannot be correctly segmented, the segmentation result can be used as the over-segmentation result. Usually, the corresponding retrieval is completed by calculating the matching degree between the image features of the 2D object and the projected image features of the 3D model in the library. Therefore, the retrieval process will mainly involve the following main issues: how to unify the 2D object and the 3D model into the same representation form and analyze the features; what matching mechanism is used for efficient retrieval?

The viewpoint coordinates are converted to spherical coordinates, and binary 〈*ϕ*, *θ*〉 is used to represent the deflection angle of viewpoint relative to the model, *ϕ* is used to represent the horizontal deflection angle, and *θ* is used to represent the rigid deflection angle

(2) $$\begin{array}{c} \phi =\arctan\frac{\sqrt{X_{E}^{2}+Y_{E}^{2}}}{Z_{E}^{2}},\\ \theta =\frac{\pi }{2}-\arctan\frac{Y_{E}}{X_{E}}. \end{array}$$

**Figure 1:**
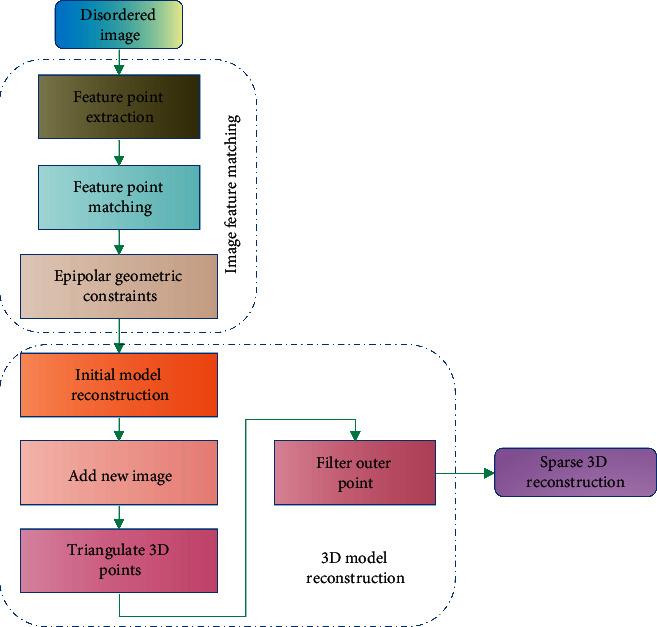
Basic flow chart of sparse TDR.

To keep all two-dimensional models consistent with the structure of the object to be built, all scene observation viewpoints should be projected under this viewpoint. The basic process of sparse point cloud reconstruction based on incremental TDR is depicted in Figure 1, which is divided into two parts: image feature matching, camera pose recovery, and spatial structure recovery.

To begin, geometric constraints are used to filter out mismatches in the initial feature point matching pairs. Basic and essential matrices are two of the most common geometric constraints. Second, two images must be chosen in order to reconstruct the initial model. The use of two good images for initialization is crucial in the TDR algorithm. The global optimal solution will not be found if the initialization falls into the local minimum. By performing a beam adjustment on the model reconstructed from the two initial images, the initial model can be made more accurate. Because of noise, calculation errors, and certain errors in image feature matching, errors in camera pose estimation and 3D coordinate restoration occur in incremental TDR every time a new image is added. The cumulative error increases as the number of images increases, resulting in poor TDR results.

Assuming that the number of images participating in TDR is *n*, and *C*_*i*_ is the internal participation and external parameter of the *i* th image, *m* three-dimensional space points are reconstructed, the coordinate of the *j* th three-dimensional space point is *X*_*j*_, and the objective function optimized by the beam adjustment method is

(3) $$\begin{array}{c} g\left(C,X\right)=\sum_{i=1}^{n}\sum_{j=1}^{m}w_{ij}{\left\|q_{ij}-P\left(C_{i},X_{j}\right)\right\|}^{2}, \end{array}$$

where *w*_*ij*_ is the indicator variable, which represents whether point *j* exists in image *i*, if point *j* is in image *i*, *w*_*ij*_ is 1; otherwise, it is 0. *P*(*C*_*i*_, *X*_*j*_) is the coordinate of point *j* on image *i* after projection transformation, and *q*_*ij*_ is the actual image coordinate of point *j* on image *i*.

In practice, Levenberg–Marquardt algorithm is usually used to iteratively optimize the minimum reprojection error [10], which is the most widely used nonlinear least square algorithm, and its iterative formula is as follows:

(4) $$\begin{array}{c} \Delta =-{\left(J_{f}^{T}J_{f}+\lambda I\right)}^{-1}J_{f}^{T}f, \end{array}$$

where *λ* is the weight parameter; when *yλ* is large, the above formula is the gradient descent method with a small step size, and when *λ* is small, it is the Gauss-Newton method.

The process of solving the optimal value is that after one iteration, when the objective function drops successfully, the value of *λ* will be reduced; otherwise, the value of *λ* is increased and iterated again. After several iterations, the optimized parameter variables can be output when the error value is less than a certain threshold.

### 3.2. Research on 3D Indoor Scene Layout

In reality, it is common for people to need to decorate a new room when they move in. Sofas, coffee tables, televisions, tables, chairs, and benches are frequently arranged in disarray at this time, and people frequently want to know how to arrange these items to create a comfortable, beautiful, simple, and generous new home. There are some established rules to guide experienced designers on how to lay out furniture, such as in a living room, how to lay out furniture to make it easier for people sitting in different positions to talk, how to consider the room layout reasonable, visual balance, and compactness.

**Figure 2:**
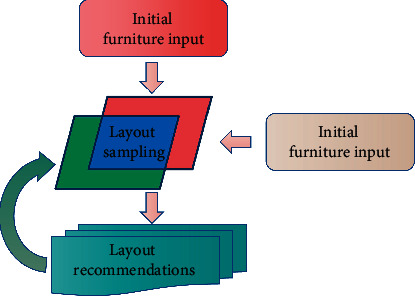
Method is not intended.

The input of this chapter is the furniture model randomly placed by users. Based on the functional rules such as pairing and conversation and visual rules such as balance and neatness, a series of reasonable furniture layout suggestions are obtained through Markov chain Monte Carlo sampling. The flow of the whole algorithm is shown in Figure 2. Next, the algorithm flow of each stage is briefly introduced.

The layout proposal is generated by sampling through the density function, which associates the defined rules and represents the layout state of the furniture collection in the current space. The sampling method adopts the Markov chain Monte Carlo method to optimize and display different furniture layout suggestions through iteration and state transition.

The functional rules of furniture layout are based on human physiological constraints and the influence of spatial layout on human behavior. The statistics of human physical characteristics, such as body size and shape, are called anthropometry. Conversation comfort is often affected by the relative placement of seats, and strong factors such as sight, body orientation, and speaking distance all greatly affect conversation comfort. People's normal walking is similar. It requires that the layout can make a person walk around the room unimpeded without being affected.

For many furniture, it is necessary to reserve a certain space around them so that people can walk to the furniture or make the furniture work normally. The furniture onto a two-dimensional plane is projected, and the constraint space to Minkowski sum of furniture projection is added, thus defining a set of formulas *J*_*F*_ that describe the areas of individual furniture and surrounding space. We use *C*_*cv*_(*I*) to minimize the overlapping area of the surrounding space of different furniture.

(5) $$\begin{array}{c} C_{cv}\left(I\right)=\sum_{f,g\in J_{F}\cup \left\{\bar{R}\right\}}A\left(f\cap g\right). \end{array}$$

Among them, *A*(·) represents the area operator, $\bar{R}$ represents the polygon area of the room, and *J*_*F*_ is estimated from the area of the whole room.

The following are the methods for optimizing and designing indoor color using VR technology: the entire interior scene of a home is modeled, as well as individual models of each room, and then presented in a three-dimensional model. First and foremost, two-dimensional graphics must be collected and input, which is done using cameras. Following the creation of the two-dimensional model, the computer converts it into the corresponding three-dimensional model. The 3D model is then given a second color design and a corresponding light rendering. The final step in creating a 3D indoor scene is to improve the details of all types of furniture. Other objects can be used as an aid to help the 3D indoor scene achieve a more realistic simulation effect, assuming the number of models has been fixed.

*u* is defined as the distance from floodlight to plane, *k* is the radius of attenuation distance of long-distance light, and *g* is the corresponding display lamp distance. If there is a line segment *n* perpendicular to the light source on the *x* axis, and the corresponding number of light sources is *m*, then the corresponding illumination brightness is obtained according to the following formula:

(6) $$\begin{array}{c} W\left(x_{j}\right)=\sum_{j=1}^{m}W_{j}\left(x_{j},\rho_{j}\right). \end{array}$$

The simulation effect corresponding to indirect light is obtained, and the lighting feeling with perfect color brightness in indoor home design is realized.

In order to facilitate more convenient and intuitive color matching, different furnishings and furniture of indoor homes can be colored by establishing different material units to ensure more flexible color matching and higher efficiency. The materials are relatively independent, and they can be superimposed on each other to ensure the richness of colors. The material unit can be widely applied to tables, chairs, sofas, pendants, doors, and windows in indoor home design.

Then, geometric modeling of indoor object models is carried out by using laser holographic images. After mapping and color simulation of the built models, the 3D indoor scenes and models are further optimized to form a 3D layout planning model of example scenes that can be displayed by laser holographic images [8].

Because the RGB⁃D image sequence is not an accurate result in the layout prediction of new layout instances, there are certain layout conflicts, such as the phenomenon that the functional areas across the wall and the functional areas overlap [10]. Therefore, in the layout planning of 3D indoor scenes, the occlusion edges in RGB⁃D images are calculated, mainly including no wall penetration, no overlap, and no door blocking. The constraint conditions are set as follows:

(7) $$\begin{array}{c} E=\sum_{i=1}\frac{d\left(a_{1},a_{2},\cdots ,a_{n}\right)}{A}, \end{array}$$

where *a*_1_, *a*_2_, ⋯, *a*_*n*_ represents wall-crossing constraint, door-blocking constraint, and overlapping constraint, respectively; ∑_*i*=1_*d* represents the description parameter of layout information.

When planning a path for a more complex indoor environment, it is necessary to browse and switch the indoor environment, determine the path to be planned, select the navigation type that can be used for path planning, and select the key position in the indoor environment and the key position of the data in the holographic image. Then, the set related service parameters are imported into the navigation service item, and the expression state of the result is finally determined so that the holographic image can perfectly express the 3D indoor scene [16].

## 4. Results Analysis and Discussion

Indoor point cloud reconstruction based on VR technology is an important basic work in this paper. A key improvement point on this issue is the combination of real-time reconstruction and off-line reconstruction. In off-line reconstruction, feature matching and incremental reconstruction processes are enhanced for the prior information of position, and attitude is obtained through real-time reconstruction.

**Figure 3:**
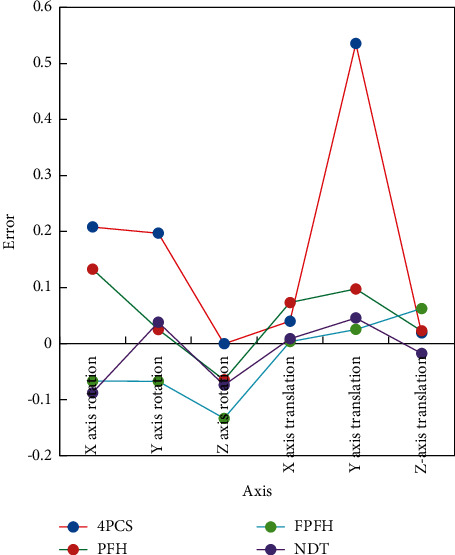
Error analysis.

In this paper, four algorithms, 4PCS (4-points congruent sets), PFH (point feature histogram), FPFH (fast point feature histograms), and NDT (normal distribution transform), are used to test two pieces of indoor point cloud data, respectively. At the same time, in order to enhance the comparability, the parameters set by these four algorithms are all the optimal parameters. After running in vs2017 + pcl environment, the result is shown in Figure 3.

From the error analysis in Figure 3, it can be seen that the 4PCS algorithm divides the original two disordered point clouds into upper and lower layers, and the initial attitude of the target point cloud has been well corrected, but the stability of the algorithm is not strong. Through a large number of parameter adjustments, this paper finds that the above-mentioned effects are random, and the generated results will be different every time the program is run. Comparing PFH and FPFH algorithms, although the basic principles of these two algorithms are similar, they can correct the attitude of the target point cloud.

Compared with 4PCS algorithm and NDT algorithm, it is obviously much higher, so it is not suitable for the real-time registration and splicing of point clouds in 3D indoor scenes, which makes it unsuitable for the real-time construction of 3D indoor scenes. Through the above analysis, it can be concluded that although the grid parameters of the NDT algorithm are the key to its registration accuracy, its high registration accuracy and high running efficiency will become the preferred coarse registration algorithm in the construction of 3D indoor scenes.

**Figure 4:**
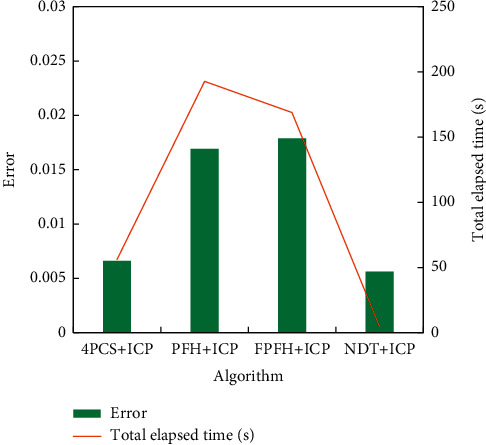
Algorithm time-consuming and error analysis.

Through ICP (iterative closest point) fine matching operation, the positions of the point clouds to be matched have been greatly adjusted, and they are more coincident with the corresponding points. As mentioned earlier, a good initial position is the key to determine ICP fine matching.  Figure 4 shows the algorithm's time-consuming and error analysis.

It can be seen from Figure 4 that the FPFH algorithm and PFH algorithm are not suitable for the real-time construction of 3D indoor scenes in terms of registration accuracy or running efficiency. Although the registration accuracy of the 4PCS + ICP algorithm is high, when the overlapping area of two point clouds is small, this algorithm runs unsteadily and takes a relatively long time. Experiments show that the NDT + ICP algorithm is obviously superior to other algorithms in registration accuracy and running efficiency, and it is effective for point clouds of 3D indoor scenes.

The original point cloud scale represents the number of 3D points in the reconstructed dense point cloud, the preprocessed point cloud scale represents the number of 3D points in the point cloud after downsampling, statistical denoising, and smooth resampling, the number of candidate wall faces represents the number of candidate wall faces after plane fitting, plane merging, and plane classification, and the number of reconstructed wall faces represents the number of wall faces in the final room layout after optimal solution.

**Figure 5:**
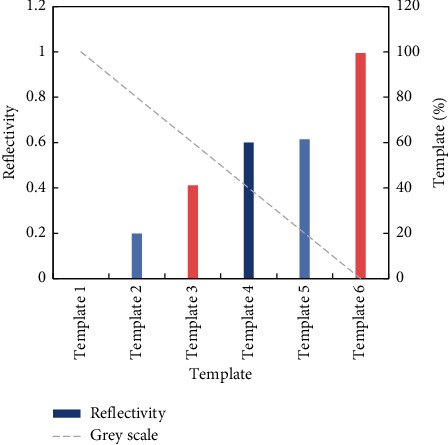
Reflectivity of different gray levels.

In order to establish the model of the relationship between reflectivity and depth values captured by different types of sensors, this paper designs and utilizes strip plane templates with six different gray levels. The gray value of the plane is divided into six levels, and the reflectivity of each level is shown in Figure 5.

**Figure 6:**
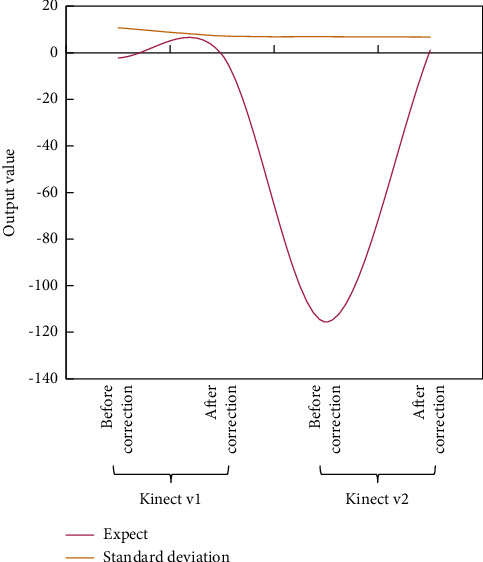
Expected value and standard deviation.

**Figure 7:**
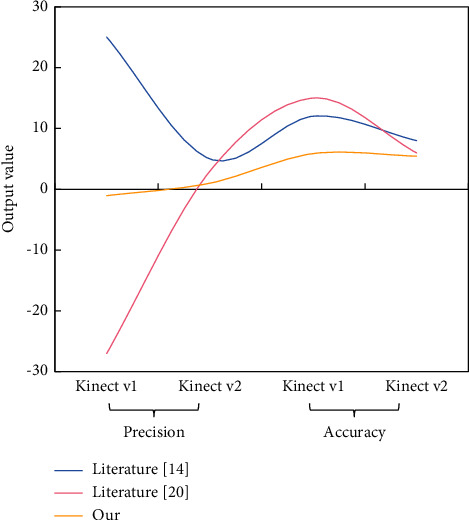
Comparative result.

The proposed neural network-based model is trained by optimizing the polynomial logistic regression objective using the RMSprop algorithm. There are five hidden layers in both Kinect v1 and Kinect v2. This procedure entails adjusting various parameters on a regular basis in order to achieve the desired results. The test set contains 260 data points, based on the above distribution ratio because the total data set used in this paper is 1300. The experimental results are shown in Figures 6 and 7.

Using this more than intelligent postcorrection neural network, for Kinect vl, depth accuracy is increased by 1.1 mm and depth accuracy by 4.3 mm. Meanwhile, for Kinect v2, the depth accuracy is increased by 115.6 mm, and the depth accuracy is increased by 0.3 mm. However, the author of [12] used the most advanced correction method to obtain the depth accuracy of its report. In addition, the results of the previous method are compared with those of this method, and it is found that the accuracy and precision of this method are better than those of the previous methods.

The room is encircled by white walls, storage cabinets, and glass walls, among other things. Many weak textures and repetitive and nondiffuse reflection environments present significant challenges for the motion recovery structure algorithm. Although the visual inertia odometer results are used for spatial local matching, cumulative drift errors are introduced into the incremental motion recovery structure algorithm, causing wall reconstruction artifacts.

**Figure 8:**
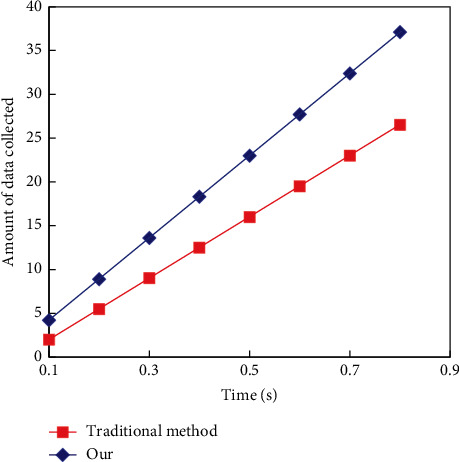
Comparison of effectiveness in data collection.

The experiment will compare the data collection effects of the two methods in 3D indoor. The two methods not only collect the overall conditions of 3D doors and windows, buildings, and rooms but also collect the details of indoor modules, floors, and network structures, which can more comprehensively reflect the advantages and disadvantages of the two methods in data collection.  Figure 8 shows the comparison of the two experimental methods in data collection.

According to the comparison results in Figure 8, it can be seen that the proposed method has a better effect in indoor data collection. It mainly applies a variety of key algorithms to optimize and analyze indoor-related data and also carries out unique data collection ideas according to the characteristics of data, which effectively improves the efficiency of data collection.

**Figure 9:**
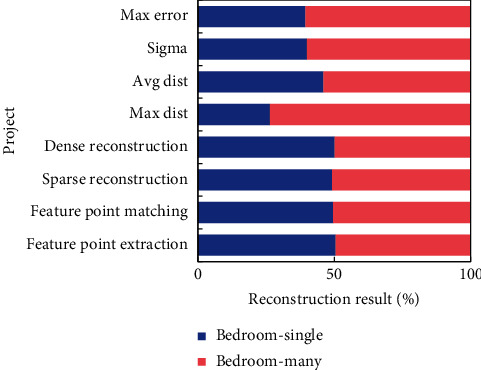
Reconstruction result of bedroom scene after removing dynamic objects.

The relative motion between the static object in the scene and the camera is caused by the camera's motion, whereas the motion of the dynamic object in the scene is affected by both the camera's relative motion and its own motion at the same time, so the motion of each dynamic object differs from the motion of the static object in the scene. The number of moving objects in the image influences the number of static and dynamic feature points, and the number of dynamic and static feature points influences the estimation of camera internal and external parameters. The effect of the number of moving objects in the scene on the TDR algorithm is discussed using various numbers of moving objects. The bedroom group's experimental data were subjected to the same TDR experiment, with 380 images in total. Three experiments were carried out, including 90 single moving targets, 90 multiple moving targets, and no moving targets. Detailed reconstruction data results are shown in Figure 9.

The experimental scenes are the living room and bedroom. Each scene is set with three modes, namely, no dynamic objects, single dynamic objects, and multidynamic objects. TDR is carried out before and after removing dynamic objects. From the experimental results, it can be seen that the reconstruction effect after removing dynamic objects is better than that without removing dynamic objects, and the reconstruction effect of single dynamic objects is better than that of multidynamic objects. In the 3D target test of the reconstructed point cloud model, more accurate recognition results can be obtained.

In the aspect of the indoor effect, not only the indoor path is optimized but also the effects such as details coloring and details perfection are carried out on the already built model. However, the traditional method lacks the adjustment of details, which makes the layout planning of 3D indoor scenes too monotonous to immerse users in images.

## 5. Conclusion

A digital model of indoor landscape reconstruction can be created using VR technology. Dense TDR is realized in a 3D indoor scene using an RGB-D sensor, and real-time operation is achieved for an extended period of time using only the CPU. Experiments show that when the initial position and pose of the two point clouds are good, the ICP algorithm is ideal, and there is no problem with finding the local optimal solution. Experiments show that, based on the characteristics of indoor point clouds, the NDT + ICP registration scheme can achieve ideal registration and mosaic results in terms of time and accuracy, which can be used to construct 3D scenes in real time. A layout recommendation method based on the Markov chain Monte Carlo sampling method can complete diversified furniture layout recommendations quickly and efficiently with little interaction. However, based on the current research, the model deformation method will be introduced into the modeling process, which will be changed according to the model's own style, in order to obtain a layout result that is more realistic with the actual scene.

## Data Availability

The data used to support the findings of this study are included within the article.
